# A robust and rapid xenograft model to assess efficacy of chemotherapeutic agents for human acute myeloid leukemia

**DOI:** 10.1038/bcj.2015.19

**Published:** 2015-03-20

**Authors:** E Saland, H Boutzen, R Castellano, L Pouyet, E Griessinger, C Larrue, F de Toni, S Scotland, M David, G Danet-Desnoyers, F Vergez, Y Barreira, Y Collette, C Récher, J-E Sarry

**Affiliations:** 1 Cancer Research Center of Toulouse, INSERM, U1037, Toulouse, France; 2 University of Toulouse, Toulouse, France; 3 Cancer Research Center of Marseille, INSERM, U1068, Marseille, France; 4 Institut Paoli-Calmettes, Marseille, France; 5 Aix-Marseille Université, Marseille, France; 6 CNRS, UMR7258, Marseille, France; 7 Centre Méditerranéen de Médecine Moléculaire, INSERM, U1065, Nice, France; 8 Université de Nice Sophia Antipolis, Nice, France; 9 Department of Medicine, University of Pennsylvania School of Medicine, Philadelphia, PA, USA; 10 Service d'Expérimentation Animale, UMS006, 31059, Toulouse cedex, France; 11 Département d'Hématologie, Centre Hospitalier Universitaire de Toulouse, Institut Universitaire du Cancer Toulouse Oncopole, Toulouse cedex, France

## Abstract

Relevant preclinical mouse models are crucial to screen new therapeutic agents for acute myeloid leukemia (AML). Current *in vivo* models based on the use of patient samples are not easy to establish and manipulate in the laboratory. Our objective was to develop robust xenograft models of human AML using well-characterized cell lines as a more accessible and faster alternative to those incorporating the use of patient-derived AML cells. Five widely used AML cell lines representing various AML subtypes were transplanted and expanded into highly immunodeficient non-obese diabetic/LtSz-severe combined immunodeficiency IL2Rγ_c_^null^ mice (for example, cell line-derived xenografts). We show here that bone marrow sublethal conditioning with busulfan or irradiation has equal efficiency for the xenotransplantation of AML cell lines. Although higher number of injected AML cells did not change tumor engraftment in bone marrow and spleen, it significantly reduced the overall survival in mice for all tested AML cell lines. On the basis of AML cell characteristics, these models also exhibited a broad range of overall mouse survival, engraftment, tissue infiltration and aggressiveness. Thus, we have established a robust, rapid and straightforward *in vivo* model based on engraftment behavior of AML cell lines, all vital prerequisites for testing new therapeutic agents in preclinical studies.

## Introduction

Acute myeloid leukemia (AML) is the most common adult acute leukemia and is characterized by clonal expansion of immature myeloblasts, initiating from rare leukemic stem or progenitor cells. In Europe and the USA, the incidence and mortality rates of AML are about 5 to 8/100.000 and 4 to 6/100.000 per year, respectively.^1^ Despite a high rate of complete remission after treatment with genotoxic agents, the relapse rate remains very high and the prognosis very poor. Overall survival at 5 years is ~30–40% in patients younger than 60 years, and <20% in patients over 60 years. Front-line chemotherapy is highly effective in ablating leukemic cells, but distant relapses are observed in the majority of patients, characterized by a refractory phase during which no other treatment has shown any efficacy thus far. Relapses are caused by malignant cell regrowth initiated by chemoresistant leukemic clones.^2, 3^

This unfavorable situation leads to a strong need for new therapeutic strategies, as well as relevant preclinical mouse models in which to test them. We have previously established a robust xenotransplantation model to study primary human AML biology and stem cell function based on the engraftment of primary AML samples into non-obese diabetic (NOD)/LtSz-severe combined immunodeficiency (SCID) IL2Rγ_c_^null^ (NSG) mice.^4, 5^ These highly immunodeficient mice have the advantages of a longer life span and higher levels of engraftment of human AML cells compared with other immunocompromised mouse strains such as SCID, NOD-SCID.^4, 6, 7^ However, this model is based on three-to-six month-long experiments with a large excess of cells and requires access to a biobank of diverse primary AML patient samples. Moreover, extensive characterization of this xenograft model with more commonly used and easily accessible AML cell lines has not been reported to date in the same setting to compare models *in vivo*. Herein, we report experimental conditions (bone marrow preconditioning and injected cell number) for a faster and easier preclinical mouse model of AML based on the xenotransplantation of a panel of six adult and childhood human AML cell lines, representing different FAB types, genetic variants and chromosomal abnormalities. In addition, we have also shown that our cell line-derived xenograft (CLDX as mirrored to patient-derived xenograft) models exhibited a broad range of overall mice survival, engraftment, tissue infiltration and aggressiveness based on their AML cell line characteristics.

## Materials and methods

### Cell lines and culture conditions

Human AML cell lines U937, MV4-11, MOLM-14, HL-60 (DSMZ, Braunschweig, Germany) and KG1a (ATCC, Manassas, VA, USA) were maintained in minimum essential medium-α medium supplemented with 10% fetal bovine serum (Invitrogen, Carlsbad, CA, USA). All cell lines were grown in the presence of 100 units per ml of penicillin and 100 μg/ml of streptomycin, and were incubated at 37 °C with 5% CO_2_. The cultured cells were split every 2–3 days and maintained in an exponential growth phase. All cell lines were annually re-ordered to DSMZ or ATCC stocks and periodically authenticated by morphologic inspection and mutational sequencing, and tested negative for Mycoplasma. The clinical and mutational features of our AML cell lines are described in Table 1.

### Flow cytometric analysis

CD45-APC (BD, San Jose, CA, USA; 555485), CD33-PE (BD 555450), CD44-FITC (BD 555478), CD44-PE-Cy7 (BD 560533), CD45.1-PerCP-Cy5.5 (BD 560580), CD34-APC-Cy7 (Biolegend, Ozyme, Saint Quentin Yvelines, France; 343514), Lin1-FITC (BD 340546), CD123-PE (BD 340545), CD45RA-Alexa Fluor 700 (BD 560673) and CD38-APC (BD 555362) fluorescent antibodies were used to analyze leukemic cells before and after injection into animals to determine phenotypic analysis of engrafted cells and percentage of leukemic cell engraftment. Absolute cell counts were determined with CountBright absolute counting beads (Invitrogen) following the manufacturer's recommendations.

### Xenotransplantation of human leukemic cells

Animals were used in accordance with a protocol reviewed and approved by the Institutional Animal Care and User Ethical Committee of the UMS006 and Région Midi-Pyrénées (Approval#13-U1037-JES08). NSG mice were produced at the Genotoul Anexplo platform at Toulouse (France) using breeders obtained from The Charles River Laboratory. Mice were housed in sterile conditions using high-efficiency particulate arrestance filtered micro-isolators and fed with irradiated food and acidified water.

Adult mice (6–8 weeks old) were sublethally irradiated with 250 cGy of total body irradiation or treated with 20 mg/kg busulfan (Busilvex, Pierre Fabre, France) by intraperitoneal administration 24 h before injection of leukemic cells. Cultured AML cell lines were washed twice in phosphate-buffered saline (PBS) and cleared of aggregates and debris using a 0.2-mm cell filter, and suspended in PBS at a final concentration of 0.2–2 million cells per 200 μl of PBS per mouse for intravenous injection. Xenograft tumors were generated by injecting AML cells (in 200 μl of PBS) in the tail vein of NSG mice. Daily monitoring of mice for symptoms of disease (ruffled coat, hunched back, weakness and reduced motility) determined the time of killing for injected animals with signs of distress.

### Hematopoietic cells count

Peripheral blood was obtained with retro-orbital bleeding. Fifty microliter of blood were collected in heparin-coated collection tube for analyzed with hematology counter (ABX Micros 60, Horiba, Montpellier, France).

### Assessment of leukemic engraftment

NSG mice were humanely killed in accordance with the Institutional Animal Care and User Ethical Committee of the UMS006 and Région Midi-Pyrénées Protocols. Bone marrow (mixed from tibias and femurs) and spleen were dissected, crushed in PBS and made into single cell suspensions for analysis by flow cytometry (FACS Calibur, FACS Canto, FACS LSR II–BD Biosciences, San Jose, CA, USA).

### Statistical analysis

Mann–Whitney test was used to calculate final *P*-values. Significance is represented as followed: *P<0.05, **P<0.01 and ***P<0.005.

## Results and discussion

The objective of this study was to develop robust xenograft models by establishing the specific experimental conditions (cell dose, dissemination tropism, growth kinetics, symptoms, lethality and stability of cell surface markers) required for the evaluation of therapeutic agents in routine and easy-to-use setting. The advantages of using well-established AML cell lines as compared with primary patient samples are the unlimited access to a large amount of human AML cells and a faster engraftment in immunodeficient mice. We analyzed *in vivo* the five most commonly used AML cell lines with a range of molecular abnormalities, clinical, biological and immunophenotypical characteristics (Table 1). MOLM-14 and MV4-11 cells are a widely studied model for FLT3-ITD AML (30% of AML patients associated with worst prognosis). U937 cells are a model for monocytic development and translocation t(10;11)(p13;q14) leading to PICALM-MLLZ10/AF10 fusion gene found in 7% of AML patients with good prognosis factor. HL-60 cells are a model for human myeloid promyelocytic cell differentiation and proliferation, and KG1a is a model for immature myeloid progenitor phenotype and FGFR1 kinase fusion genes (Table 1). These cells were transplanted intravenously in adult NSG mice. Importantly, AML cell lines kill mice as does AML in patients, whereas primary AML cells in NSG mice rarely kill the animals.

### Busulfan or irradiation conditioning step does not change the xenotransplantation efficacy of AML cells

**Figure 1:**
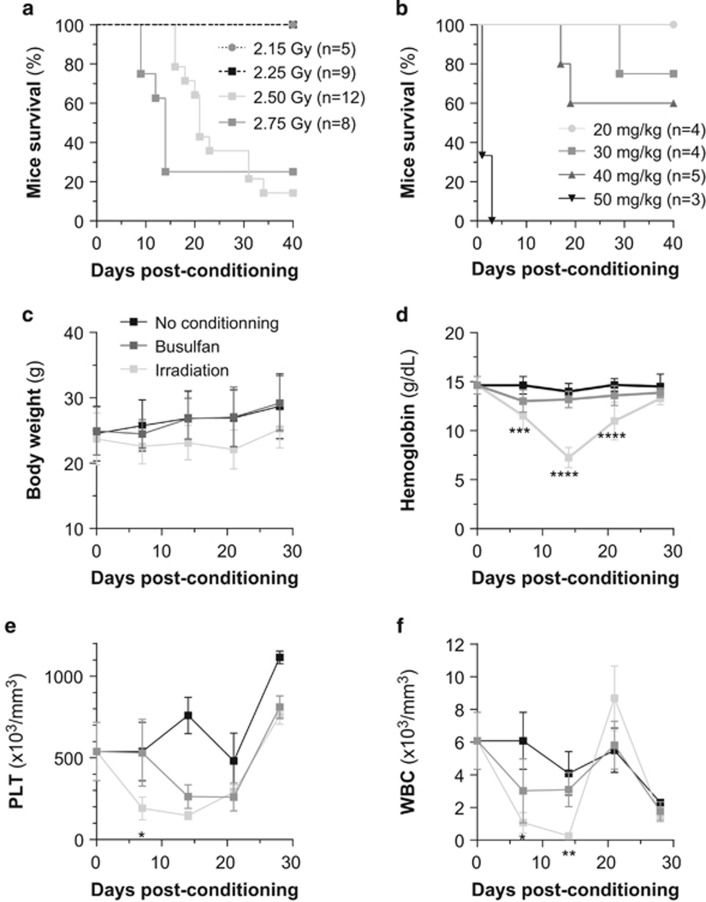
Impact of various bone marrow conditioning treatments on the overall and hematological toxicity and the engraftment efficacy of AML cell lines in highly immunodeficient NSG mice. Kaplan–Meyer curve of the mice overall survival during the maximal tolerated dose study (**a**, with irradiation dose escalade; **b**, with busulfan dose escalade). Adult NSG mice non-conditioned (*n*=5) or irradiated at 2.25 Gy (*n*=5) or treated with 20 mg/kg busulfan (*n*=5) were weighed (**c**) and their blood was collected each week post conditioning for assays of hemoglobin (**d**), platelets (**e**) and white blood cells (**f**).

Xenotransplantation of primary human AML or normal CD34^+^ cells is greatly enhanced if recipients receive a chemical conditioning regimen such as busulfan (a myeloablative alkylating agent, 25–35 mg/kg) or total body irradiation (up to 4 Gy).^8, 9, 10, 11^ However, it is not clear whether conditioning improves the engraftment of human AML cell lines especially in the most recently developed NSG mouse model. We first performed a maximally tolerated dose study to define the best conditioning procedures (Figures 1a and b) and then tested the effect of the determined sublethal conditions (20 mg/kg busulfan vs 2.25 Gy irradiation) on NSG mice. Body weight, hemoglobin, platelet number and white blood cell number were monitored over 4 weeks following the conditioning procedure. Irradiation severely impacts mice body weight and blood counts, whereas busulfan injection has a milder effect on these parameters (Figures 1c and f). In both the procedures, normal mouse hematopoiesis is recovered 3 weeks post conditioning.

**Figure 2:**
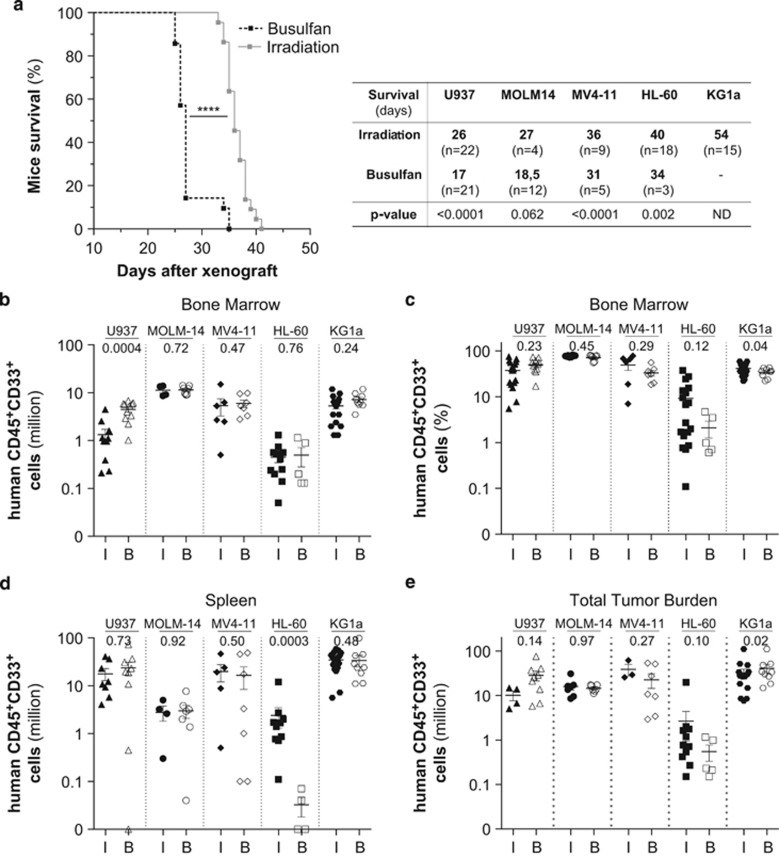
Impact of the conditioning methods on the xenotransplantation of different AML cell lines in highly immunodeficient NSG mice. Adult NSG mice were injected with 2 × 10^6^ of different AML cells (HL-60, MOLM-14, MV4-11, U937 and KG1a) 24 h post conditioning (I, irradiation at 2.25 Gy; B, 20 mg/kg busulfan). Mice survival is analyzed with Kaplan–Meyer curve (**a**) and the engraftment level is assessed by flow cytometry in bone marrow (million, **b**; percent, **c**), spleen (million, **d**) and in bone marrow+spleen (total cell tumor burden, **e**).

We observed a significant reduction in overall survival of the xenografted mice in the busulfan conditioning setting as compared with irradiation setting (Figure 2a) but observed no significant difference in leukemic engraftment level in bone marrow and spleen or total cell tumor burden after transplantation of four out the five AML (U937, KG1a, MOLM-14 and MV4-11) cell lines tested (Figures 1b and e), as previously observed in NOD-SCID for human cell engraftment. ^11^

### AML cell lines exhibit a broad range of engraftment, tissue infiltration and aggressiveness

**Figure 3:**
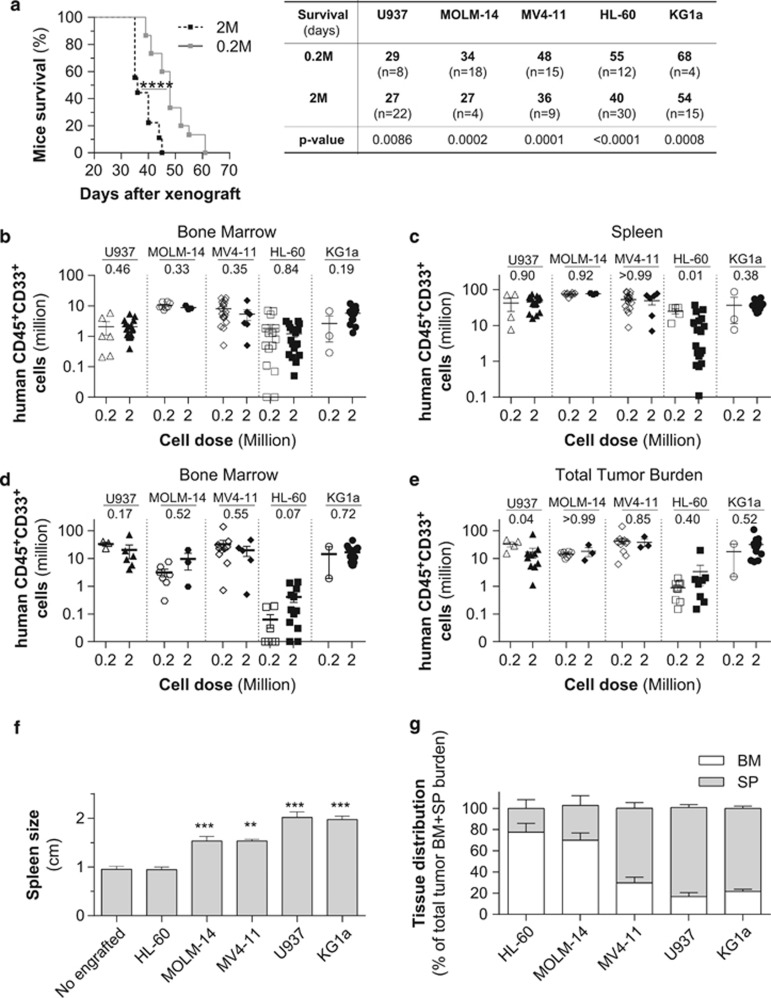
Impact of the injected cell dose on the xenotransplantation of different AML cell lines in highly immunodeficient NSG mice. NSG mice were injected with two doses (0.2 or 2 Millions) of different AML cells (HL-60, MOLM-14, MV4-11, U937 and KG1a). Mice survival was analyzed after xenotranplantation (**a**). The engraftment level was evaluated in the bone marrow (million, **b**; percent, **c**) and spleen (million, **d**), as well as the total tumor cell burden in hematopoietic mice organs (million, **e**). Tumor invasion in hematopoietic organs was shown through the spleen size (**f**) and the tissue distribution in hematopoietic organs (**g**). BM, bone marrow; SP, spleen. **P<0.01 and ***P<0.005.

A recurrent problem in leukemic adoptive transfer is the issue of the cell dose injected, to avoid either engraftment failure or delay or, on the contrary, a tumor burden outgrowth leading to a premature animal death precluding any investigation. Thus, we asked whether the cell number injected into NSG mice would affect the overall mice survival. For 0.2 million injected AML cells, recipient mice survival was dependent upon the AML cell line and ranged from 28 to 68 days (U937<MOLM-14<MV4-11<HL-60<KG1a; Figure 3a). As expected, increasing the injected cell number (up to 2 m) decreased the overall mice survival for all AML cell lines tested (Figure 3a). For both doses, U937 cells appeared to be the most aggressive AML cell line, paralyzing and killing the mice within 4 weeks, whereas mice engrafted with KG1a cells had the longest overall survival with a median of 54 and 68 days for 2 m versus 0.2 m cells injected, respectively (Figure 3a).

The variability in mice survival between cell lines was not associated with differences in leukemic cells infiltration in the bone marrow (Figures 3b and c), spleen (Figure 3d) or the total tumor burden (Figure 3e), regardless of the injected leukemic cell dose. Accordingly, decreasing injected cell number increases the time to engraft without changing the engraftment capacity at the dissection time and has the disadvantage of extending the *in vivo* efficacy assay. Furthermore, we noted a variable total cell tumor burden in hematopoietic tissues (bone marrow and spleen) depending on the AML cell line type (2 m for HL-60; 9 m, U937; 12 m for MOLM-14; 13 m, KG1a; and 30 m MV4-11; Figure 3e). The engraftment level of HL-60 cells was lowest compared with other cell lines. MV4-11 cells have the highest expansion level in hematopoietic tissues of xenografted mice. Although most of the AML cell lines invade the spleen and exhibit a spleenomegaly, HL-60 and MOLM-14 cells are preferentially located in the bone marrow (Figures 3f and g), indicating differential tissue tropism for this panel of AML cell lines.

These results show a diversity of engraftment capacities and aggressiveness in these AML cell lines, as well as a diversity in tissue tropism, consistent with what we observed with AML patient cells transplanted in the same immunodeficient mice strain.^4^ Engraftment levels, tissue distribution and overall survival are not associated with any clinical features and cytogenetic abnormalities. Interestingly, we also observed that AML cell lines bearing differentiation markers such as U937, MOLM-14 and MV4-11 appeared to have a more aggressive *in vivo* behavior than immature like AML cell lines such as HL-60 or KG1a.

### The overall immunophenotype of AML cells is conserved *in vivo*

**Figure 4:**
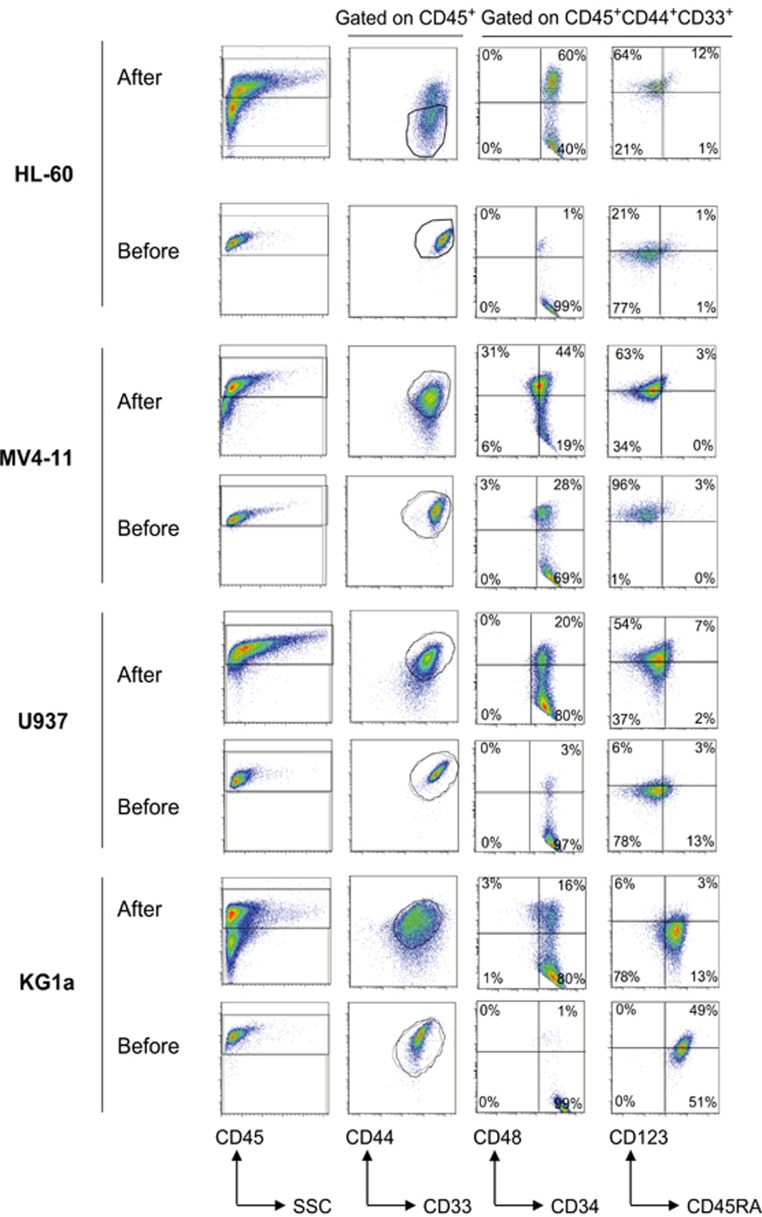
Analysis of the expression of major myeloid cell surface markers in AML cell lines before and after xenotransplantation. The immunophenotype of HL-60, MV4-11, U937 and KG1a cells was analyzed on BD LSRII Fortessa Flow Cytometer using human CD45, CD44, CD33, CD34, CD38, CD45RA and CD123 before and after xenotransplantation in our NSG mice model. All AML cell lines are SSC^low^, CD45, CD44 and CD33 positive *in vitro* and *in vivo*.

We next addressed whether the immunophenotype of AML cell lines was conserved *in vivo*. The expression level of various myeloid cell surface markers such as CD45, CD33, CD44, CD34, CD38, CD45RA and CD123 was analyzed for four AML cell lines before and after xenotransplantation (Figure 4). Most of those markers appeared globally unchanged during engraftment, however, we found that the transplantation of HL-60 cells in mice also led to the appearance of a second population CD44^dim^ (Figure 4, second column). Moreover, the frequency of CD34^+^CD38^+^ was significantly increased *in vivo* to the detriment of the CD34^+^CD38^-^ population (Figure 4, third column). With the sole exception of the CD38 marker, the NSG-based model of AML maintains cell phenotype more consistently than in the NOD-SCID model.^12^

Here we provide necessary technical details and conditions that other laboratories may use to quickly and routinely set up *in vivo* AML experiments. A xenografts based on native or engineered cell lines are not novel, this is the first study that compares the five most well-characterized AML cell lines in the same experimental settings and mouse strain. The establishment of this preclinical AML model is of special relevance and significance to drug-sensitivity studies as *in vitro* cell culture-based screens do not accurately reflect *in vivo* effects and responses. In conclusion, we have found that the xenotransplantation models of five well-characterized AML cell lines (for example, KG1a, HL-60, MOLM-14, MV4-11 and U937) exhibited a broad range of mice survival, engraftment, tissue infiltration and aggressiveness. By increasing or decreasing the number of injected leukemic cells, we can modulate mice survival without changing the tissue distribution of leukemic cells and their engraftment in NSG mice. This work has also shown that AML cell lines kill mice in a manner similar to that as patients in clinics but different from the xenograft of primary AML patient specimens in NSG mice. Moreover, in contrast to patient-derived xenograft models, these CLDX models are a powerful, rapid and straightforward *in vivo* assay available to all leukemia research laboratories to perform preclinical studies to assess the *in vivo* efficacy of conventional or targeted therapies in AML.

## Figures and Tables

**Table 1:** Clinical, mutational and biological features of AML cell lines used in this *in vivo* study

*Name*	*Gender*	*FAB*		*Karyotype*	*Model for*	*FLT3*		*NPM1*	*IDH1* *R132*	*IDH2* *R140*	*IDH2* *R172*	*DNMT3A*	*CEBPa*	*Kit*	*NRas*	*KRas*	*WT1*	*p53*	*c-Myc*	*PTEN*
						*ITD*	*TKD*													
KG1a	M	M0	Relapse	Complex	FGFR1OP2-FGFR1	wt	wt	wt	wt	wt	wt	wt	wt	wt	+	wt		Mutated	Overexpressed	Mutated
HL-60	F	M2	Dx	Complex		wt	wt	wt	wt	wt	wt	wt	wt	wt	+	wt	+	Null; deleted	Amplified	wt
MV4-11^a^	M	M5	Dx	Complex	MLL-AF4 w/FLT3-ITD	ITD	wt	wt	wt	wt	wt	wt		wt	wt	wt		wt	Overexpressed	wt
MOLM14	M	M5	Relapse	Complex	MLL-AF4 w/FLT3-ITD	ITD	wt	wt	wt	wt	wt	wt	wt	wt	wt	wt	+	wt	Overexpressed	wt
U937	M	M5	Refractory	t(10;11)(p13;q14)	CALM-AF10	wt	wt	wt	wt	wt	wt	wt	wt	wt	wt	wt	+	Null; deleted	Overexpressed	Null

Abbreviations: F, female; M, male; wt, wild type.

a Childhood.
